# Cocoa by-products extracts suppress viral replication and oxidative stress in chikungunya virus-infected cells

**DOI:** 10.1371/journal.pone.0354240

**Published:** 2026-07-22

**Authors:** Sandra Johanna Morantes, Luisa Fernanda Daza, Alejandro Cáceres, Luis Felipe Gutiérrez, Martha Cecilia Rincón, Juan David Dereix, Félix Giovanni Delgado

**Affiliations:** 1 Semillero de Investigación en Bioprospección & Biotecnología Farmacéutica, Programa Química Farmacéutica, Universidad El Bosque, Bogotá, Colombia; 2 Grupo de Investigación en Química Aplicada – INQA, Programa Química Farmacéutica, Universidad El Bosque, Bogotá, Colombia; 3 Grupo de Virología, Vicerrectoría de Investigaciones, Universidad El Bosque, Bogotá, Colombia; 4 Grupo de Investigación en Biomoléculas Alimentarias, Instituto de Ciencia y Tecnología de Alimentos, Universidad Nacional de Colombia Sede Bogotá, Bogotá, Colombia; 5 MetCore - Metabolomics Core Facility, Vice-Presidency for Research, Universidad de los Andes, Bogotá, Colombia; Universidad Cooperativa de Colombia, COLOMBIA

## Abstract

**Context:**

Studies have demonstrated a correlation between oxidative stress and Chikungunya virus (CHIKV) replication, underscoring the potential of these biochemical interactions for developing novel antiviral therapies. Since cocoa (*Theobroma cacao* *L.*) by-products have exhibited potent antiviral and antioxidant properties, they may represent a promising source for new therapeutic alternatives.

**Objective:**

To assess the potential of cocoa pod husk (CPH) and cocoa bean shell (CBS) extracts to inhibit viral replication and oxidative stress in an *in vitro* model of CHIKV infection.

**Materials and methods:**

The viability of Huh-7 cells in the presence of extracts (15.6 to 250 µg/mL), and CHIKV-infected cells treated with the extracts was evaluated using the resazurin reduction assay. Antiviral activity was determined by RT-qPCR and plaque assays, while reduction of oxidative stress in infected cells was measured using the dichloro-dihydro-fluorescein diacetate (DCFH-DA) assay at 12 and 24 hours post-infection (hpi).

**Results:**

CPH and CBS extracts exhibited minimal cytotoxicity in Huh-7 cells. Remarkably, both extracts exhibited antiviral and antioxidant properties at concentrations above 62.5 µg/mL mainly at 12 hpi. This was evidenced by a significant reduction in viral RNA copies and infectious virus particles, as well as decreased levels of reactive oxygen species (ROS) in CHIKV-infected cells.

**Discussion and conclusion:**

Cocoa extracts reduce the oxidative stress induced by CHIKV in Huh-7 cells while exhibiting antiviral properties, highlighting their potential to modulate both oxidative stress and viral replication. Further studies are recommended to elucidate the mechanisms by which these extracts work.

## Introduction

Chikungunya fever is an arthropod-borne viral disease transmitted to humans by *Aedes aegypti* and *Aedes albopictus* mosquitoes infected with the chikungunya virus (CHIKV) [1]. According to the International Committee on Taxonomy of Viruses (ICTV), CHIKV is a positive-sense, single-stranded RNA virus classified within the family *Togaviridae* and the genus *Alphavirus* [2]. Structurally, CHIKV is an enveloped virus composed of a lipid bilayer containing the E1 and E2 glycoproteins, which mediate viral entry into host cells. The envelope surrounds an icosahedral capsid formed by the capsid (C) protein, which encloses the viral genome of approximately 11.8 kb. The genome is organized into two open reading frames (ORFs), separated by a non-coding junction and flanked by two untranslated regions. The 5′ ORF encodes four non-structural proteins (nsP1, nsP2, nsP3, and nsP4) involved in viral replication, whereas the 3′ ORF encodes five structural proteins, including the capsid (C), E3, E2, 6K, and E1, which are essential for virion assembly and infectivity [2,3].

Since the first reported case of chikungunya fever in Tanzania in 1952, the virus has progressively spread across Africa, Asia, and Europe, eventually reaching the Americas by late 2013. In 2025, a total of 485,908 cases of CHIKV disease and 229 associated deaths have been reported worldwide, representing an increase compared with the same period in 2024 [4]. Clinically, the disease is characterized by an abrupt onset of symptoms, including fever, severe polyarthralgia and myalgia, headache, fatigue, nausea, skin rash, and conjunctival injection, which typically manifest within 2–7 days following infection [1]. Moreover, a significant proportion of infected individuals develop chronic manifestations collectively referred to as post-chikungunya rheumatism, a long-term condition with clinical features resembling rheumatoid arthritis [5]. Despite its global distribution and substantial public health impact, current clinical management remains largely supportive and is limited to symptomatic treatment with analgesics, anti-inflammatory drugs, adequate hydration, and rest, which underscores the persistent burden of the disease and highlights the urgent need for effective antiviral strategies [1].

The interplay between cellular oxidative stress and CHIKV infection is a critical determinant of disease pathogenesis in humans and is closely associated with excessive production of reactive oxygen species (ROS) [6]. This redox imbalance promotes oxidative damage of essential macromolecules, including lipids, proteins, and DNA, ultimately disrupting key cellular processes that facilitate viral replication and disease progression [7]. In this context, the Huh7 hepatoma cell line is widely employed as an *in vitro* model for CHIKV infection due to its high permissiveness to RNA viruses and its suitability for investigating virus-induced oxidative stress [8,9]. Supporting this approach, Mishra et al, systematically mapped direct interactions between the CHIKV nonstructural protein nsP3 and host proteins in Huh7 cells identifying 52 statistically significant host interacting partners. Based on these findings, the authors proposed a working model in which CHIKV-mediated perturbation of host protein networks disrupts mitochondrial homeostasis, leading to enhanced ROS production and altered cellular energy metabolism. These mitochondrial alterations favor viral replication while simultaneously impairing antiviral signaling through the mitochondrial antiviral-signaling protein (MAVS) [10]. Collectively, these observations highlight host–virus interaction pathways as promising therapeutic targets to limit virus-induced cellular damage and inhibit viral replication.

The exploration of natural products as alternative therapeutic options for viral diseases has intensified in recent years [11]. Among these, cacao fruit and its processing by-products have attracted increasing attention due to their rich and diverse composition of bioactive compounds, including catechins, epicatechins, procyanidins, theobromine, caffeine, and other secondary metabolites, which exhibit antioxidant, antibacterial, and antiviral activities [12–14]. Previous studies have demonstrated the antiviral potential of cacao-derived compounds against several RNA viruses; notably, Kamei et al. reported that a cocoa extract exerted dose-dependent antiviral activity *in vitro* and *in vivo* against human influenza A (H1N1, H3N2), influenza B, and avian influenza viruses (H5N1, H5N9) [15]. More recently, Guimarães Santana et al. identified two *Theobroma cacao* cystatins (TcCYS3 and TcCYS4) capable of binding SARS-CoV-2 proteases, resulting in reduced viral genomic copy numbers [16]. Despite this evidence, the antiviral activity of cacao-derived extracts against CHIKV has not yet been explored. Importantly, phenolic-rich cacao extracts have been shown to enhance cellular antioxidant defenses and attenuate oxidative damage in multiple *in vitro* and *in vivo* models, findings further supported by human studies linking cacao polyphenol intake to improved oxidative balance [17,18]. Given that CHIKV infection is strongly associated with oxidative stress and excessive ROS production, these antioxidants properties provide a strong rationale for investigating cacao-derived extracts as potential modulators of CHIKV-induced pathogenic mechanisms.

Considering the pharmacological potential of bioactive compounds present in the main cacao by-products, the aim of this study was to evaluate the antiviral activity of two phenolic-rich extracts obtained from cocoa pod husk (CPH) and cocoa bean shell (CBS) against CHIKV in Huh-7 cells. In addition, we investigated whether the antiviral effects of these extracts could be associated with their ability to modulate CHIKV-induced oxidative stress, thereby providing mechanistic insight into their potential role in attenuating virus-driven pathogenic process.

## Materials and methods

### Reagents

Resazurin (R7017), 2,7-dichlorodihydrofluorescein diacetate, DCFH-DA (D6883), reagents for preparing RIPA buffer: 50 mM Tris HCl pH 7.4, 150 mM NaCl, 1.0% (v/v) NP-40, 0.5% (w/v) sodium deoxycholate, 1.0 mM EDTA, 0.1% (w/v) SDS: sodium dodecyl sulfate, phosphate-buffered saline (PBS), and Hank’s balanced salt solution (HBSS), 2,4,6 TPTZ, sodium acetate trihydrate, ferric chloride hexahydrate, and gallic acid (> 98%), carboxymethylcellulose sodium salt (CMC) were provided by Sigma-Aldrich, St. Louis, MI, USA. Dimethyl sulfoxide (DMSO) was supplied by AppliChem (USA). DPPH 99%, and 6-hydroxy-2,5,7,8-tetramethyl chroman-2-carboxylic acid (Trolox, ≥ 97%) and Folin-Ciocalteu (2N) reagent were supplied by Merck (Germany).

### Cocoa extracts preparation

The CPH and the CBS (variety TCS01), were provided by Agrosavia (Centro de Investigación El Mira (1.5512257,-78.7007294), Nariño, Colombia). Both materials were separately ground using a hammer mill and sieved prior to obtaining ethanolic extracts by microwave-assisted extraction, as previously described [19]. Briefly, powders of CPH and CBS of average diameter of 100 µm, were mixed with ethanolic solutions (63% v/v) at a liquid to solid ratio 67:1 mL/g and extracted in a microwave environment of 400 W power 2400 GHz, during 240 and 185 s, respectively. The extracts were filtered through a Whatman filter paper No. 4, concentrated and dried under vacuum and stored in dark bottles at −40°C, until analyzed. The stock solution of the extracts was prepared in a 1:1 mixture of DMSO and PBS at a concentration of 50 mg/mL. Working solutions were prepared in culture medium with 5 serial 1:2 dilutions (250, 125, 62.5, 31.25, and 15.6 µg/mL). The final DMSO concentration in the wells did not exceed 0.1%.

### Fourier transform infrared spectroscopy (FTIR) analyses

FTIR spectroscopy was employed as a non-invasive technique to examine the presence of functional groups present in the dried ethanolic extracts obtained from CBS and CPH. FTIR analyses were recorded using a FT/IR-4700 infrared spectrophotometer (JASCO Corp., Tokyo, Japan), equipped with ATR (single horizontal reflection diamond prism). Spectra were analyzed as described previously [20] using OriginPro 2021 software in the spectral range 4000–600 cm ^−^ ^¹^ with 24 scans recorded at a resolution of 4 cm ⁻ ¹.

### Determination of total phenolic content (TPC)

The TPC was determined according to the Folin-Ciocalteu colorimetric assay, as reported by Sanchez-Reinoso et al [20]. The extracts (500 μL) were dissolved in 30 mL of distilled water into a volumetric flask (50 mL) and mixed with 500 μL of the Folin–Ciocalteu reagent. After 2 min, Na_2_CO_3_ 20% w/v (2 mL) was added, and the volume was adjusted to 50 mL with distilled water. The absorbance at 765 nm was read after 2 h using a UV-Vis Spectrophotometer V-530 (JASCO Corp., Tokyo, Japan). Distilled water was used as a reaction blank. Gallic acid (Merck, Germany) was used as standard (0.031–1.0 mg/mL; R^2^ = 0.998). Results were expressed as mg gallic acid equivalent (GA) per gram of CPH or CBS on a dry basis (mg GA/g CPH or CBS).

### DPPH and FRAP assays

The antioxidant capacity of both CPH and CBS extracts was determined by means of radical scavenging 2,2-diphenyl-1-picrylhydrazyl (DPPH) and the ferric ion reducing antioxidant power (FRAP) assays, according to the methods described in previous publications [21–23]. Briefly, for the DPPH assay, 3 mg of DPPH were dissolved in 80 mL of methanol, by mixing for 30 min. Subsequently, 1950 μL of this methanolic DPPH solution were combined with 50 μL of extract, and the samples were left to rest for 1 h, protected from light and at room temperature. The absorbance was measured using a UV-Vis spectrophotometer V-530 (JASCO Corp., Tokyo, Japan) at a wavelength of 517 nm, with methanol as blank. For the FRAP assay, a solution containing 20 mM FeCl₃, 300 mM acetate buffer at pH 3.6 (prepared with 3 g of CH₃COONa and 16 mL of CH₃COOH), and a solution of TPTZ (2,4,6-tris(2-pyridyl)-s-triazine) were prepared in a ratio of 1:10:1, respectively. In a microcell, 450 μL of this solution was mixed with 20 μL of extract and 735 μL of distilled water, which was then left to rest for 30 min, protected from light and at room temperature. Its absorbance was measured at 593 nm using a UV-Vis spectrophotometer V-530 (JASCO Corp., Tokyo, Japan).

The results for each assay were calculated from standard Trolox solution calibration curves at concentrations of 0.15–1 mM, except for the FRAP assay for CBS, where the curve ranged from 0.0625–1 mM. Each assay was performed in triplicate, and the results were expressed as μmol TE/g of extract (CPH or CBS).

### Chemical composition analysis by Reversed-Phase Liquid Chromatography Quadrupole Time-of-Flight Mass Spectrometry (RP-LC-QTOF-MS)

Five milligrams of each lyophilized extract were suspended in 500 μL of methanol (MeOH), vortex-mixed for 10 min at 3200 rpm, followed by sonication for 10 min, and then centrifuged at 13,000 rpm at 4 °C for 10 min. The resulting supernatants were used for liquid chromatography – mass spectrometry (LC–MS) analysis. Chromatographic separation was performed using an Agilent Technologies 1290 Infinity LC system coupled to a quadrupole time-of-flight MS (QTOF 6545, Agilent Technologies) equipped with an electrospray ionization (ESI) source. An aliquot of 2 μL of each extract was injected onto a C18 column (InfinityLab Poroshell 120 EC-C18, 100 × 2.1 mm, 1.9 μm) maintained at 30 °C. Separation was achieved using a gradient elution consisting of 0.1% (v/v) formic acid in Milli-Q water (mobile phase A) and 0.1% (v/v) formic acid in acetonitrile (mobile phase B), at a constant flow rate of 0.4 mL min  ^−¹^. The gradient started at 2% B, increased linearly to 30% B over 10 min, followed by an increase to 98% B at 20 min, maintained for 2 min, then returned to initial conditions and equilibrated for 5 min. MS detection was carried out in GNPS-compatible AutoMS/MS mode over an m/z range of 50–1700 in both positive and negative ionization modes. Continuous mass calibration was performed using reference ions at m/z 121.0509 (C_5_H_4_N_4_) and m/z 922.0098 (C_18_H_18_O_6_N_3_P_3_F_24_) in positive ion mode, and m/z 112.9856 [C_2_O_2_F_3_(NH_4_)] and m/z 1033.9881 (C_18_H_18_O_6_N_3_P_3_F_24_) in negative ion mode.

LC–MS raw data were processed using Agilent MassHunter Profinder software version 10.0 (B.10.0, Agilent Technologies) for molecular feature extraction, including deconvolution, alignment, and integration. Metabolite annotation was performed using MS-DIAL version 5.1 and the CEU Mass Mediator annotator (http://ceumass.eps.uspceu.es), incorporating exact mass matching, theoretical formula generation based on isotopic distributions, verification of retention times, and evaluation of adduct formation. Metabolite identification confidence was assigned according to the Metabolomics Standards Initiative (MSI) guidelines (levels 1–4), using spectral matching against public databases including the MS-DIAL spectral library, GNPS, MassBank, and ReSpect. To reduce background and contaminant signals, a blank filtering step was applied, retaining only those features whose peak area in the samples was at least fivefold higher than the corresponding area detected in blank injections.

### Cell lines and growth conditions

The adherent Huh-7 human cell line (JCRB0403; CVCL_0336) derived from a liver tumor of a 57-year-old Japanese male, and VERO cell line (ATCC_CCL-81; CVCL_0059) established from the kidney of a normal adult African green monkey (*Chlorocebus sabaeus*), were cultured in RPMI 1640 medium (L0495, Biowest) and DMEM high glucose medium (D6429, Sigma) respectively, supplemented with 10% fetal bovine serum (FBS; S1810, Biowest) and 25 µg/mL gentamicin (G1264, Sigma). Cells were propagated in T-75 cm^2^ culture flasks at 37°C in a 5% CO_2_ atmosphere with 90% relative humidity.

### Virus and infection conditions

CHIKV was kindly provided by Professor Eliana Calvo (Universidad El Bosque, Bogotá, Colombia) and corresponds to the CHIKV-G464 strain, also known as COL7624 (GenBank ID: MH329298.1). The characterization of this viral isolate has been previously described [24]. As shown by Archila et al, the full genome was obtained by next-generation sequencing. Infective and replicative capacities were evaluated in HEK 293T/17 (ATCC_ CRL-11268G-1; CVCL_UE07) a human embryonic kidney cell line stably expressing the organic anion transporter 1 (OAT1/SLC22A6), Huh-7 (JCRB0403; CVCL_0336), and MRC-5 (ATCC CCL-171; CVCL_0440), a human lung fibroblast cell line established from embryonic tissue. Infection rates and cytopathic effects were assessed by flow cytometry and resazurin fluorescence assay, respectively. Viral titers were determined by plaque assay, and viral protein and genomic RNA kinetics were analyzed by western blot and RT-qPCR, respectively [24].

For infection assays, Huh-7 cells were seeded in 96-well plates at a density of 30,000 cells/well. After 24 h, cells were infected with a multiplicity of infection (MOI) of 0.1 (calculated based on number of plaque-forming units (PFUs) per cell) for 90 min. An uninfected mock control was included (Mock). After viral adsorption, the inoculum was removed, and replaced with fresh medium containing vehicle, or extracts. Supernatants and cells monolayers were collected at 12 and 24 hpi, time point selected according to the previously described replication kinetics of the same CHIKV isolate, which represent early and intermediate stages of infection before extensive cytopathic damage develops [24]. Supernatants were stored frozen to quantify the effect of treatments on viral replication and plaque formation. The cell monolayer was used to analyze cell viability and ROS production.

### Cell viability assay

Huh-7 cell viability was evaluated under three experimental conditions: (i) cells treated with cacao-derived extracts, (ii) CHIKV-infected cells, and (iii) CHIKV-infected cells treated with the extracts. Cell viability was assessed indirectly by evaluating metabolic activity using the resazurin reduction assay, which is based on the ability of viable and metabolically active cells to convert the non-fluorescent compound resazurin into the fluorescent product resorufin. In addition, an evaluation of the cell monolayer was performed using phase contrast microscopy. For assay, cells were seeded in 96 well flat-bottom plates at a density of 30,000 cells/well. After allowing cell adhesion, cells were exposed for 24 h to five serial 1:2 dilutions of the extracts (15.6–250 µg/mL). A 25% (v/v) DMSO solution was used as a positive control for cell death. For the vehicle control, cells received an equivalent volume of a 1:1 (v/v) DMSO: PBS solution adjusted to match the final DMSO concentration present at the highest extract concentration tested. In all experimental conditions, the final DMSO concentration did not exceed 0.1% (v/v). The concentration range evaluated in this study was selected according to the criteria established by the U.S. National Cancer Institute (NCI) for the cytotoxic classification of crude extracts [25,26]. Since extracts with IC₅₀ values between 201 and 500 µg/mL are considered weakly cytotoxic, 250 µg/mL was chosen as the highest concentration tested, representing an intermediate value within this range. For viability experiments involving CHIKV infection, either alone or in combination with extract treatment, the previously described infection conditions were applied. After removing the virus, 100 µL of the extracts (15.6–250 µg/mL) were added, and viability was evaluated at 12 and 24 hpi, since longer incubation resulted in a cytopathic effect due to CHIKV. Following the exposure period, supernatants were collected, and 100 µL of 44 µM resazurin were added to each well. After a 4 h incubation, the fluorescence emitted by viable and/or metabolically active cells was quantified using a spectrofluorometer (Infinite M200pro, TECAN) at an excitation wavelength of 530 nm and an emission wavelength of 590 nm. Wells containing only the assay reagent without cells were included to measure baseline fluorescence generated by the reagent itself. This background signal was subtracted from all fluorescence readings obtained from experimental wells prior to data analysis. The assays were performed in three independent experiments, each in triplicate. Results were reported as percentages of viability relative to the vehicle control.

### RNA purification and one-step RT-qPCR

Collected supernatants were used for RNA extraction, which was performed with the QIAamp Viral RNA Mini Kit (Qiagen), according to the manufacturer’s instructions. Purified RNA was tested using RT-qPCR with a primer-probe set targeting the nsp4 region of the CHIKV genome (prototype strain S27, GenBank accession no. NC_004162), as described previously [27]. Briefly, amplification was carried out in a final volume of 15 μL using the Luna Universal Probe One-Step RT-qPCR Kit (NEB), with 5 μL of RNA template (60–80 ng/μL), 0.4 μM of probe, and 0.4 μM of each primer. The amplification conditions were as follows: 15 min at 55°C, 3 min at 95°C, followed by 15 seconds at 95°C, 30 seconds at 55°C, and 15 seconds at 72°C for a total of 39 amplification cycles. Absolute quantification of genomic copies was determined using a calibration curve generated with a control plasmid that was designed in the laboratory.

### CHIKV quantification by plaque assays

Vero cells were seeded in 24-well plates at a density of 4 × 10^4^ cells per well in 500 µL of DMEM supplemented with 10% fetal bovine serum (FBS) and 1% antibiotic (penicillin-streptomycin). Twenty‑four hours later, the cells were infected for 1 h at 37°C in a humidified atmosphere containing 5% CO_2_ using 10-fold serial dilutions of supernatants collected at 12 and 24 hpi from Huh-7 cells infected with CHIKV and treated with either CPH or CBS extracts. After infection, without removal of the viral inoculum, the cell monolayers were overlaid with 800 µL of semi-solid medium supplemented with 3.3% FBS and 1% carboxymethylcellulose sodium salt (CMC) (Sigma–Aldrich, St. Louis, MO, USA). After 72 h incubation, the monolayers were fixed and stained with 1% crystal violet. Viral titer was presented as PFU per mL. It was calculated using the formula: Titer (PFU/mL) = (Number of plaques) x (Dilution factor) / Volume of inoculum (mL).

### Evaluation of cellular antioxidant capacity

The cellular antioxidant capacity of the cacao extracts was assessed by measuring the reduction in total intracellular ROS levels in CHIKV-infected cells using the DCFH-DA assay. The assay conditions were adapted from previous work conducted by the group and are described as follows. First, the cell monolayer was washed twice with 100 µL of PBS. Next, 100 µL of a 10 µM DCFH-DA solution in HBSS was added and the cells were incubated for 30 min. After incubation, cells were washed with PBS, and 100 µL of HBSS was added for fluorescence measurement using a spectrofluorometer (Infinite M200pro, TECAN; excitation: 485 nm, emission: 530 nm). Additionally, the cells were imaged using an Axio Vert.A1 Inverted Microscope (Carl Zeiss AG., Jena, Germany) equipped with an X-Cite 120Q series fluorescence system. Images were captured using an Axiocam 305 camera and processed using Zen 3.8 software (Carl Zeiss AG., Jena, Germany). Finally, to normalize the fluorescence data, protein was extracted from each well using RIPA buffer and quantified using the Pierce™ BCA Protein Assay Kit (Thermo Scientific). Cellular ROS levels were reported as relative fluorescence units (RFU) per µg of protein.

### Data analysis

All data were analyzed with Prism software version 10.4.1 (GraphPad Software Inc., La Jolla, CA, USA). Statistical significance was determined using the non-parametric Kruskal-Wallis test followed by Dunn’s multiple comparison test to identify differences between groups. Differences were considered significant at p < 0.05.

## Results

### The CPH and CBS extracts have antioxidant capacity

**Fig 1:**
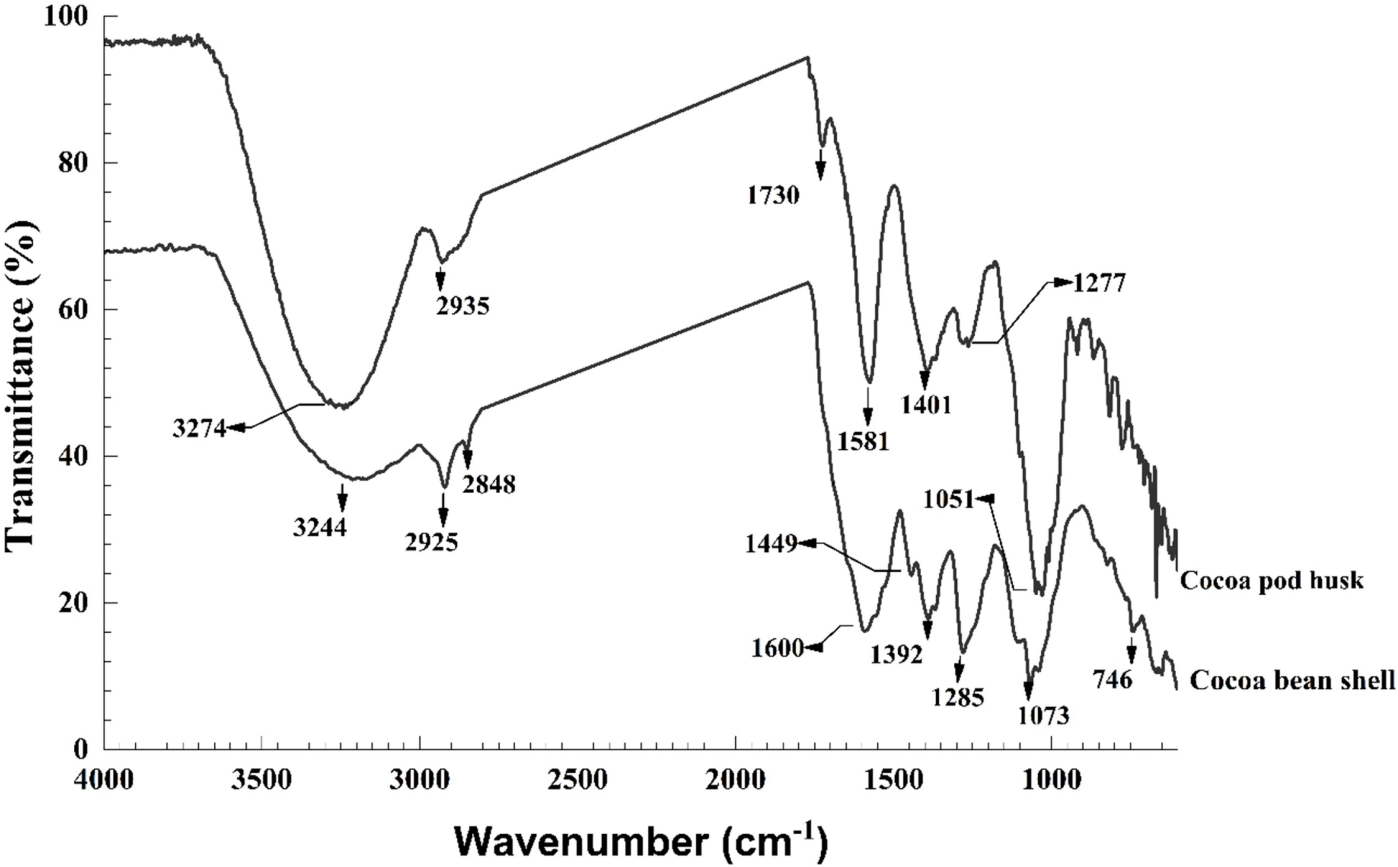
ATR-FTIR spectra of the dried ethanolic extracts obtained from cocoa pod husk (CPH) and cocoa bean shell (CBS). The graph shows transmittance (%) on the y-axis as a function of wavenumber (cm ^−^ ^¹^) on the x-axis over the range of 4000–600 cm  ^−¹^. The upper and lower spectra correspond to CPH and CBS, respectively. Arrows indicate the main absorption bands detected in each extract, and the corresponding wavenumber values are shown next to each peak. Spectra were obtained using approximately 10 mg of dried extract, with 24 scans acquired at a resolution of 4 cm ^−^ ^¹^. Differences in peak positions and intensities reflect variations in the chemical composition of the two cocoa-derived extracts.

Considering earlier findings on the antioxidant capacity of cocoa by-products extracts, we aimed to verify antioxidant activity and total phenolic content in the extracts. The total phenolic contents of the extracts obtained from CPH and CBS were 43.94 and 45.68 mg GA/g, respectively. Using a radical scavenging assay, we obtained values of 430.80 μmol Trolox equivalents (TE)/g for CPH and 341.67 μmol (TE)/g for CBS. These results demonstrate that the extracts contain bioactive compounds capable of donating electrons to the DPPH free radical confirming their ability to neutralize free radicals. Additionally, the FRAP assay yielded values of 144.32 μmol (TE)/g for CPH and 165.05 μmol (TE)/g for CBS, indicating the ability of the extracts to reduce ferric ions (Fe^3+^) to ferrous ions (Fe^2+^) demonstrating its total reducing capacity. Fig 1 shows the typical FTIR spectra for CBS and CPH extracts obtained by means of a microwave assisted extraction process. As observed, there were no significant differences between both spectra, and the extracts present typical absorption bands related to phenolic compounds and flavonoids, which are displayed in Table 1.

**Table 1:** FTIR absorption bands for cocoa by-products extracts.

Band (cm ⁻ ¹)	Assignment	Likely compounds
3274, 3244	O–H stretching (broad, typical of free hydroxyls and phenolics); possible N–H stretching (alkaloids)	Polyphenols, flavonoids, phenolic acids
2935, 2925, 2848	Aliphatic C–H stretching (CH₂/CH₃)	Fatty acids, waxes, lignin derivatives
1730	C = O stretching (esters or carboxylic acids)	Phenolic acids, lipids, natural esters
1600, 1581	Aromatic C = C stretching	Aromatic rings in flavonoids, lignin, tannins
1449, 1401, 1392	CH₂/CH₃ deformation and COO⁻ vibrations	Lignin components, carboxylic acids, aromatic compounds
1285, 1277	C–O stretching (phenols and esters); aromatic ring deformations	Flavonoids, phenolic acids
1073, 1051	C–O/C–C stretching (alcohols, carbohydrates)	Residual cellulose, phenolic compounds
746	Out-of-plane C–H bending in aromatics	Substituted aromatic rings (flavonoids, tannins)

### Chemical characterization of CPH and CBS extracts

HPLC-ESI-QTOF-MS analyses in both positive and negative ionization modes were employed to characterize the chemical composition of CPH and CBS extracts. The annotated metabolites, together with their retention times, molecular formulas, accurate masses, adducts, ionization polarity, identification levels, and relative abundances, are summarized in Tables 2 and 3. Overall, the extracts exhibited a chemically diverse profile comprising alkaloids, amino acids, carbohydrates, fatty acids and oxylipins, flavonoids, glycerophospholipids, phenylpropanoid derivatives, organic acids, peptides, sphingolipids, and terpenoids. Identification was mainly achieved at MSI level 2, based on accurate mass, isotopic pattern, and MS/MS spectral matching.

**Table 2:** Chemical Composition of CPH Analyzed by LC-MS.

Name	RT	Formula	Mass	Adduct	ID LEVEL	Area	Abundancy	Polarity
**Alkaloids**								
Theobromine	3.06	C_7_H_8_N_4_O_2_	180.0648	[M+H]^+^	2	852099	9.25	ESI^+^
Caffeine	4.91	C_8_H_10_N_4_O_2_	194.0802	[M+H]^+^	2	594234	6.45	ESI^+^
**Aminoacids**								
Proline	0.61	C_5_H_9_NO_2_	115.0637	[M+H]^+^	2	2280260	24.76	ESI^+^
Pipecolic acid	0.82	C_6_H_11_NO_2_	129.0792	[M+H]^+^	2	609173	6.61	ESI^+^
Valine	0.82	C_5_H_11_NO_2_	117.0792	[M+H]^+^	2	284940	3.09	ESI^+^
Tyrosine	1.1	C_9_H_11_NO_3_	181.074	[M+H]^+^	2	91942	1	ESI^+^
Isoleucine	1.24	C_6_H_13_NO_2_	131.0949	[M+H]^+^	2	511576	5.55	ESI^+^
Phenylalanine	2.32	C_9_H_11_NO_2_	165.0792	[M+H]^+^	2	101915	1.11	ESI^+^
Tryptophan	3.92	C_11_H_12_N_2_O_2_	204.0901	[M+H]^+^	2	13115	0.14	ESI^+^
**Carbohydrates**								
Trehalose	0.61	C_12_H_22_O_11_	342.1163	[M-H]^–^	2	1216917	1.7	ESI^-^
**Fatty acid amides**								
Oleoyl ethanolamine	19.4	C_20_H_39_NO_2_	325.2991	[M+H]^+^	3	268260	2.91	ESI^+^
**Fatty acids and oxylipins**								
Trihydroxy-octadecenoic acid	14.05	C_18_H_34_O_5_	330.241	[M-H]^–^	3	141002	0.2	ESI^-^
LFA 18:4 + 2O	14.11	C_18_H_30_O_5_	308.1992	[M-H-H2O]^–^	2	164581	0.23	ESI^-^
Linolenic acid	17.01	C_18_H_30_O_2_	278.2253	[M+H]^+^	2	354447	3.85	ESI^+^
Epoxy-octadecadienoic acid	17.51	C_18_H_30_O_3_	294.2199	[M-H]^–^	2	427157	0.6	ESI^-^
Isopalmitic acid	18.72	C_16_H_32_O_2_	256.2404	[M-H]^–^	3	118650	0.17	ESI^-^
Linoleic acid	20.14	C_18_H_32_O_2_	280.2406	[M-H]^–^	3	75317	0.1	ESI^-^
hydroxy-stearic acid	20.3	C_18_H_36_O_3_	300.2666	[M-H]^–^	2	10596	0.01	ESI^-^
**Flavonoids**								
Piperidinone	2.03	C_5_H_9_NO	99.0685	[M+H]^+^	2	154316	1.68	ESI^+^
Catechin	5.83	C_15_H_14_O_6_	290.0795	[M+H]^+^	2	104018	1.13	ESI^+^
Catechin	5.86	C_15_H_14_O_6_	290.0795	[M-H]^–^	2	181047	0.25	ESI^-^
Quercetin-arabinoside	8.11	C_20_H_18_O_11_	434.0856	[M-H]^–^	2	66169	0.09	ESI^-^
Quercetin-o-xylopyranoside	17.3	C_20_H_18_O_11_	434.2447	[M+H]^+^	2	739625	8.03	ESI^+^
**Glycerophospholipids**								
LPE 18:2	16.43	C_23_H_44_NO_7_P	477.2867	[M-H]^–^	2	27465	0.04	ESI^-^
LPC 18:2	16.53	C_26_H_50_NO_7_P	519.3342	[M+H]^+^	2	217515	2.36	ESI^+^
LPE 16:0	16.92	C_21_H_44_NO_7_P	453.2864	[M-H]^–^	2	56347	0.08	ESI^-^
LPC 16:0	17.04	C_24_H_50_NO_7_P	495.3347	[M+H]^+^	2	333373	3.62	ESI^+^
LPE 18:1	17.27	C_23_H_46_NO_7_P	479.3029	[M+H]^+^	2	32096	0.35	ESI^+^
LPE 18:1	17.27	C_23_H_46_NO_7_P	479.3025	[M-H]^–^	2	20155	0.03	ESI^-^
LPC 18:1	17.39	C_26_H_52_NO_7_P	521.3499	[M+H]^+^	2	169403	1.84	ESI^+^
LPC 18:0	18.52	C_26_H_54_NO_7_P	523.3657	[M+H]^+^	2	73347	0.8	ESI^+^
**Other phospholipids**								
Stearoylglycerophosphoinositol	18	C_27_H_53_O_12_P	600.3289	[M-H]^–^	2	60554	0.08	ESI^-^
**Jasmonates**								
Sulfooxyjasmonate	5.3	C_12_H_18_O_7_S	306.076	[M-H]^–^	2	42534	0.06	ESI^-^
**Phenylpropanoid derivatives**								
Phenylethyl-acetamide	7.78	C_10_H_13_NO	163.0997	[M+H]^+^	2	39673	0.43	ESI^+^
Phenylethanolamine	2.97	C_8_H_11_NO	121.0894	[M-H2O+H]^+^	2	10411	0.11	ESI^+^
Phenylethyl primeveroside	6.66	C_19_H_28_O_10_	416.1695	[M-H]^–^	2	2736094	3.81	ESI^-^
**Organic acids**								
Gluconic acid	0.61	C_6_H_12_O_7_	196.0581	[M-H]^–^	2	2004975	2.79	ESI^-^
Malic acid	0.63	C_4_H_6_O_5_	134.0213	[M-H]^–^	2	3070536	4.28	ESI^-^
Citric acid	0.89	C_6_H_8_O_7_	192.0262	[M-H]^–^	2	301036	0.42	ESI^-^
**Peptides and dipeptides**								
Cyclo(prolyl-valyl)	4.55	C_10_H_16_N_2_O_2_	196.1212	[M+H]^+^	2	18721	0.2	ESI^+^
**Phenolic compounds**								
Dihydroxybenzaldehyde	3.86	C_7_H_6_O_3_	138.0313	[M-H]^–^	2	165045	0.23	ESI^-^
Ferulic acid	15.27	C_10_H_10_O_4_	176.0477	[M-H2O+H]^+^	2	759271	8.24	ESI^+^
**Proanthocyanidins**								
Procyanidin B1	5.46	C_30_H_26_O_12_	578.1435	[M+H]^+^	2	136550	1.48	ESI^+^
Procyanidin B2	5.48	C_30_H_26_O_12_	578.1434	[M-H]^–^	2	167113	0.23	ESI^-^
Procyanidin C1	6.34	C_45_H_38_O_18_	866.2063	[M-H]^–^	2	160551	0.22	ESI^-^
**Sphingolipids**								
Phytosphingosine	15.57	C_18_H_39_NO_3_	317.2944	[M+H]^+^	2	374944	4.07	ESI^+^
Sphinganine	16.3	C_18_H_39_NO_2_	301.299	[M+H]^+^	2	33552	0.36	ESI^+^
**Terpenoids**								
Triterpenoid	16.92	C_30_H_46_O_3_	453.2873	[M+H]^+^	2	44951	0.49	ESI^+^

LFA: long-chain fatty acid; LPC: Lysophosphocholine; LPE: Lysophosphoethanolamine.

**Table 3:** Chemical Composition of CBS Analyzed by LC-MS.

Name	RT	Formula	Mass	Adduct	ID LEVEL	Area	Abundancy	Polarity
**Alkaloids**								
Caffeine	4.91	C_7_H_8_N_4_O_2_	194.0802	[M+H]^+^	2	17990975	9.59	ESI^+^
Theobromine	3.06	C_8_H_10_N_4_O_2_	180.0648	[M+H]^+^	2	15049048	8.02	ESI^+^
**Aminoacids**								
Isoleucine	1.24	C_6_H_13_NO_2_	131.0949	[M+H]^+^	2	23947791	12.77	ESI^+^
Phenylalanine	2.32	C_9_H_11_NO_2_	165.0792	[M+H]^+^	2	12754257	6.8	ESI^+^
Pipecolic acid	0.82	C_6_H_11_NO_2_	129.0792	[M+H]^+^	2	4549256	2.43	ESI^+^
Proline	0.61	C_5_H_9_NO_2_	115.0637	[M+H]^+^	2	4144404	2.21	ESI^+^
Tryptophan	3.92	C_11_H_12_N_2_O_2_	204.0901	[M+H]^+^	2	2099253	1.12	ESI^+^
Tyrosine	1.1	C_9_H_11_NO_3_	181.074	[M+H]^+^	2	4585552	2.44	ESI^+^
Valine	0.82	C_5_H_11_NO_2_	117.0792	[M+H]^+^	2	5508009	2.94	ESI^+^
**Carbohydrates**								
Trehalose	0.61	C_12_H_22_O_11_	342.1163	[M-H]^–^	2	540148	0.25	ESI^-^
**Fatty acid amides**								
Oleoyl ethanolamine	19.4	C_20_H_39_NO_2_	325.2991	[M+H]^+^	3	4340207	2.31	ESI^+^
**Fatty acids and oxylipins**								
Epoxy-octadecadienoic acid	17.51	C_18_H_30_O_3_	294.2199	[M-H]^–^	2	331412	0.15	ESI^-^
Hydroxy-stearic acid	20.3	C_18_H_36_O_3_	300.2666	[M-H]^–^	2	922532	0.42	ESI^-^
Isopalmitic acid	18.72	C_16_H_32_O_2_	256.2404	[M-H]^–^	3	36903	0.02	ESI^-^
LFA 18:4 + 2O	14.11	C_18_H_30_O_5_	308.1992	[M-H-H2O]^–^	2	128045	0.06	ESI^-^
Linoleic acid	20.14	C_18_H_32_O_2_	280.2406	[M-H]^–^	3	394927	0.18	ESI^-^
Linolenic acid	17.01	C_18_H_30_O_2_	278.2253	[M+H]^+^	2	879552	0.47	ESI^+^
Trihydroxy-octadecenoic acid	14.05	C_18_H_34_O_5_	330.241	[M-H]^–^	3	672763	0.31	ESI^-^
**Flavonoids**								
Catechin	5.83	C_15_H_14_O_6_	290.0795	[M+H]^+^	2	1596597	0.85	ESI^+^
Catechin	5.86	C_15_H_14_O_6_	290.0795	[M-H]^–^	2	3381206	1.55	ESI^-^
Piperidinone	2.03	C_5_H_9_NO	99.0685	[M+H]^+^	2	3983634	2.12	ESI^+^
Quercetin-arabinoside	8.11	C_20_H_18_O_11_	434.0856	[M-H]^–^	2	380240	0.17	ESI^-^
Quercetin-o-xylopyranoside	17.3	C_20_H_18_O_11_	434.2447	[M+H]^+^	2	193656	0.1	ESI^+^
**Glycerophospholipids**								
LPC 16:0	17.04	C_24_H_50_NO_7_P	495.3347	[M+H]^+^	2	11908794	6.35	ESI^+^
LPC 18:0	18.52	C_26_H_54_NO_7_P	523.3657	[M+H]^+^	2	6405378	3.41	ESI^+^
LPC 18:1	17.39	C_26_H_52_NO_7_P	521.3499	[M+H]^+^	2	29922555	15.95	ESI^+^
LPC 18:2	16.53	C_26_H_50_NO_7_P	519.3342	[M+H]^+^	2	12334692	6.58	ESI^+^
LPE 16:0	16.92	C_21_H_44_NO_7_P	453.2864	[M-H]^–^	2	1643963	0.75	ESI^-^
LPE 18:1	17.27	C_23_H_46_NO_7_P	479.3029	[M+H]^+^	2	2490576	1.33	ESI^+^
LPE 18:1	17.27	C_23_H_46_NO_7_P	479.3025	[M-H]^–^	2	2887805	1.32	ESI^-^
LPE 18:2	16.43	C_23_H_44_NO_7_P	477.2867	[M-H]^–^	2	2108606	0.97	ESI^-^
**Other phospholipids**								
Stearoylglycerophosphoinositol	18	C_27_H_53_O_12_P	600.3289	[M-H]^–^	2	1108082	0.51	ESI^-^
**Jasmonates**								
Sulfooxyjasmonate	5.3	C_12_H_18_O_7_S	306.076	[M-H]^–^	2	6640532	3.04	ESI^-^
**Phenylpropanoid derivatives**								
Phenylethyl-acetamide	7.78	C_10_H_13_NO	163.0997	[M+H]^+^	2	6765325	3.61	ESI^+^
Phenylethanolamine	2.97	C_8_H_11_NO	121.0894	[M-H2O+H]^+^	2	1629220	0.87	ESI^+^
Phenylethyl primeveroside	6.66	C_19_H_28_O_10_	416.1695	[M-H]^–^	2	65342	0.03	ESI^-^
**Organic acids**								
Citric acid	0.89	C_6_H_8_O_7_	192.0262	[M-H]^–^	2	734436	0.34	ESI^-^
Gluconic acid	0.61	C_6_H_12_O_7_	196.0581	[M-H]^–^	2	106399	0.05	ESI^-^
Malic acid	0.63	C_4_H_6_O_5_	134.0213	[M-H]^–^	2	228419	0.1	ESI^-^
**Peptides and dipeptides**								
Cyclo(prolyl-valyl)	4.55	C_10_H_16_N_2_O_2_	196.1212	[M+H]^+^	2	2510858	1.34	ESI^+^
Leucyl-Leucine	5.39	C_12_H_24_N_2_O_3_	244.1791	[M+H]^+^	2	885857	0.47	ESI^+^
Leucyl-phenylalanine	6.46	C_15_H_22_N_2_O_3_	278.1635	[M+H]^+^	2	1215530	0.65	ESI^+^
Prolylphenylalanine	7.35	C_14_H_18_N_2_O_3_	244.121	[M-H2O+H]^+^	2	888233	0.47	ESI^+^
Pyroglutamyl-Isoleucine	5.37	C_11_H_18_N_2_O_4_	242.1276	[M-H]^–^	2	625185	0.29	ESI^-^
**Phenolic compounds**								
Dihydroxybenzaldehyde	3.86	C_7_H_6_O_3_	138.0313	[M-H]^–^	2	944370	0.43	ESI^-^
Ferulic acid	15.27	C_10_H_10_O_4_	176.0477	[M-H2O+H]^+^	2	2516918	1.34	ESI^+^
Phenyllactic acid	6.46	C_9_H_10_O_3_	148.0523	[M-H-H2O]^–^	2	863285	0.4	ESI^-^
**Proanthocyanidins**								
Procyanidin B1	5.46	C_30_H_26_O_12_	578.1435	[M+H]^+^	2	729037	0.39	ESI^+^
Procyanidin B2	5.48	C_30_H_26_O_12_	578.1434	[M-H]^–^	2	1759480	0.81	ESI^-^
Procyanidin C1	6.34	C_45_H_38_O_18_	866.2063	[M-H]^–^	2	851093	0.39	ESI^-^
**Sphingolipids**								
Phytosphingosine	15.57	C_18_H_39_NO_3_	317.2944	[M+H]^+^	2	2782680	1.48	ESI^+^
Sphinganine	16.3	C_18_H_39_NO_2_	301.299	[M+H]^+^	2	1642477	0.88	ESI^+^
**Terpenoids**								
Triterpenoid	16.92	C_30_H_46_O_3_	453.2873	[M+H]^+^	2	1333827	0.71	ESI^+^

LFA: long-chain fatty acid; LPC: Lysophosphocholine; LPE: Lysophosphoethanolamine.

The CPH extract showed a high relative contribution of amino acids and organic acids (Table 2). Proline was the dominant amino acid (24.76%), followed by pipecolic acid (6.61%), isoleucine (5.55%), and valine (3.09%). Alkaloids such as theobromine (9.25%) and caffeine (6.45%) were also prominent. Organic acids, including malic acid (4.28%), gluconic acid (2.79%), and citric acid (0.42%), contributed significantly to the overall composition. Glycerophospholipids remained an important lipid class, with Lysophosphocholine (LPC) 16:0 (3.62%) and LPC 18:2 (2.36%) as the main representatives. Phenolic compounds such as ferulic acid (8.24%) and phenylethyl primeveroside (3.81%) further contributed to the chemical profile, indicating a strong presence of phenylpropanoid metabolism in this extract (Table 2).

The CBS extract presented a markedly lipid- and peptide-rich profile (Table 3). Glycerophospholipids dominated the relative abundance, particularly LPC 18:1 (15.95%), LPC 18:2 (6.58%), LPC 16:0 (6.35%), and LPC 18:0 (3.41%). Alkaloids were also highly abundant, with caffeine (9.59%) and theobromine (8.02%) among the major detected metabolites. Amino acids such as isoleucine (12.77%), phenylalanine (6.80%), tyrosine (2.44%), and valine (2.94%) contributed substantially to the nitrogenous fraction. In contrast to CPH samples, CBS showed a broader diversity of peptides and dipeptides, including cyclo(prolyl-valyl) (1.34%), leucyl-leucine (0.47%), and leucyl-phenylalanine (0.65%). Additionally, sulfooxyjasmonate (3.04%) and phenylethyl-acetamide (3.61%) were distinctive features of the CBS extract, suggesting an enhanced contribution of jasmonate-related and phenylpropanoid pathways (Table 3).

### Cocoa by-products extracts exhibited minimal cytotoxicity in Huh-7 cells

**Fig 2:**
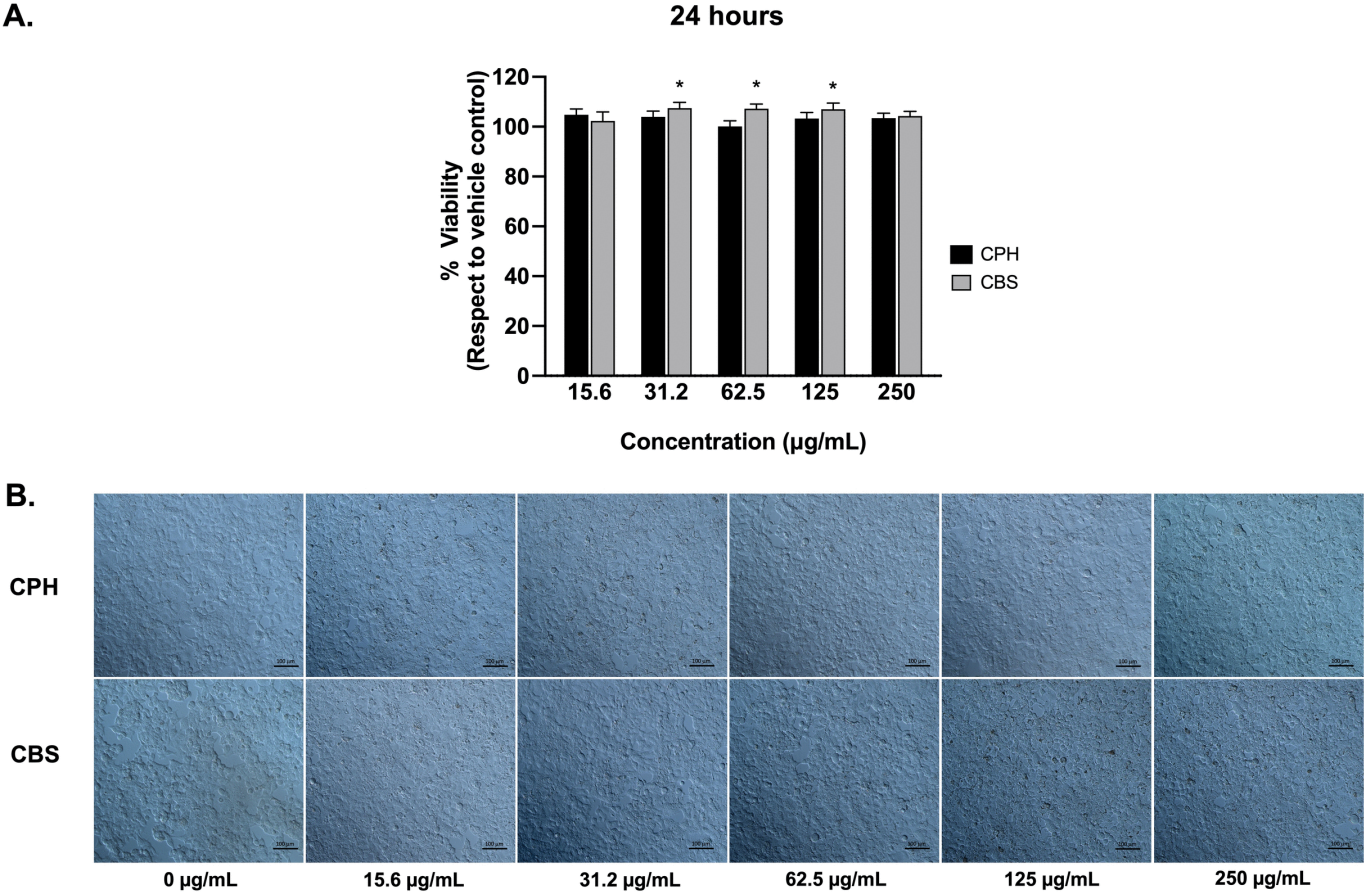
Cocoa by-products extracts exhibited minimal cytotoxicity in Huh7 cells. Huh-7 cells were treated with 15.6, 31.2, 62.5, 125 and 250 µg/mL of CPH or CBS extracts and incubated for 24 h. Then, the cells were analyzed to evaluate the cell viability using a resazurin reduction assay (A) or cell morphology using phase-contrast microscopy (scale bar 100 µm) (B). The assays were performed in three independent experiments, each in triplicate. Results were reported as percentages of viability relative to the vehicle control and are expressed as the means ± SEM. Statistical significance was determined relative to the vehicle control (*p ≤ 0.05).

Considering that the extracts could potentially influence the antiviral assay due to possible cytotoxic effects, we first analyzed the cytotoxic profile of each extract. Using five different concentrations, we monitored the cells for any metabolic or morphological changes over a 24 h incubation period. As shown in Fig 2A, the resazurin reduction assay results indicate that the extracts did not negatively impact cellular metabolic activity, as viability percentages consistently exceeded 90% at all evaluated time points (Fig 2A). However, a slight but statistically significant increase in metabolic activity was observed at CBS concentrations ranging from 31.2 to 125 µg/mL. Furthermore, the Huh-7 cells exposed to CPH or CBS extracts maintained their characteristic hexagonal epithelial morphology, and the monolayer remained adherent, with over 80% confluence across all wells (Fig 2B).

**Fig 3:**
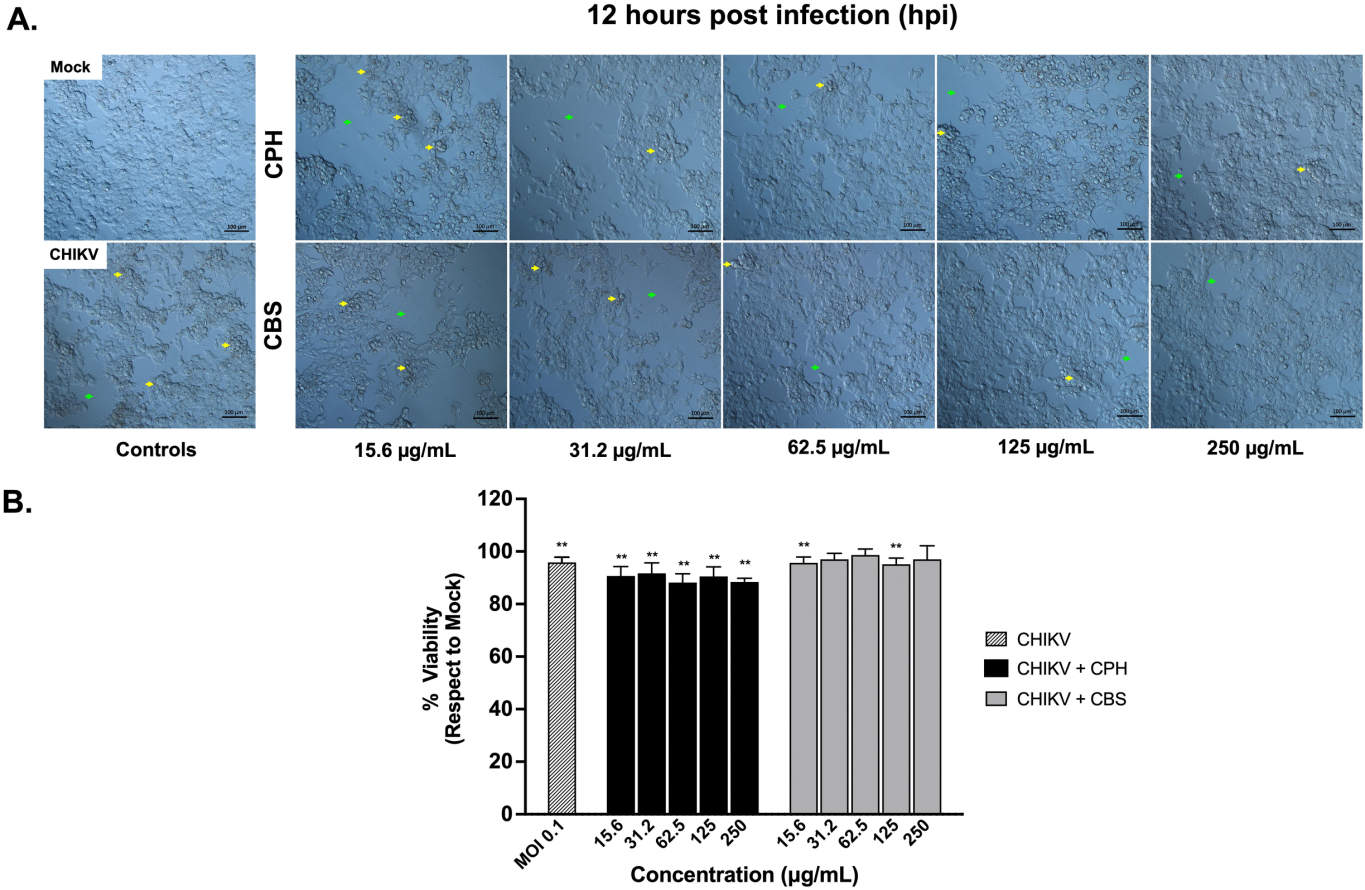
Cocoa by-products extracts attenuated morphological alterations in CHIKV-infected Huh-7 cells at 12 hpi. Huh-7 cells were infected with CHIKV at a MOI of 0.1 and then treated with 15.6, 31.2, 62.5, 125 and 250 µg/mL of CPH or CBS extracts. At 12 hpi the cells were analyzed to evaluate the cell morphology using phase-contrast microscopy (scale bar 100 µm) (A) or cell viability using a resazurin reduction assay (B). Untreated CHIKV-infected cells (CHIKV) and uninfected mock control cells (Mock) were included as experimental controls. Yellow arrows indicate virus-induced morphological alterations, whereas green arrows indicate cell detachment from the monolayer. The assays were performed in three independent experiments, each in triplicate. Results were reported as percentages of viability relative to the mock control and are expressed as the means ± SEM. Statistical significance was determined relative to the mock control (*p ≤ 0.05; **p ≤ 0.01).

**Fig 4:**
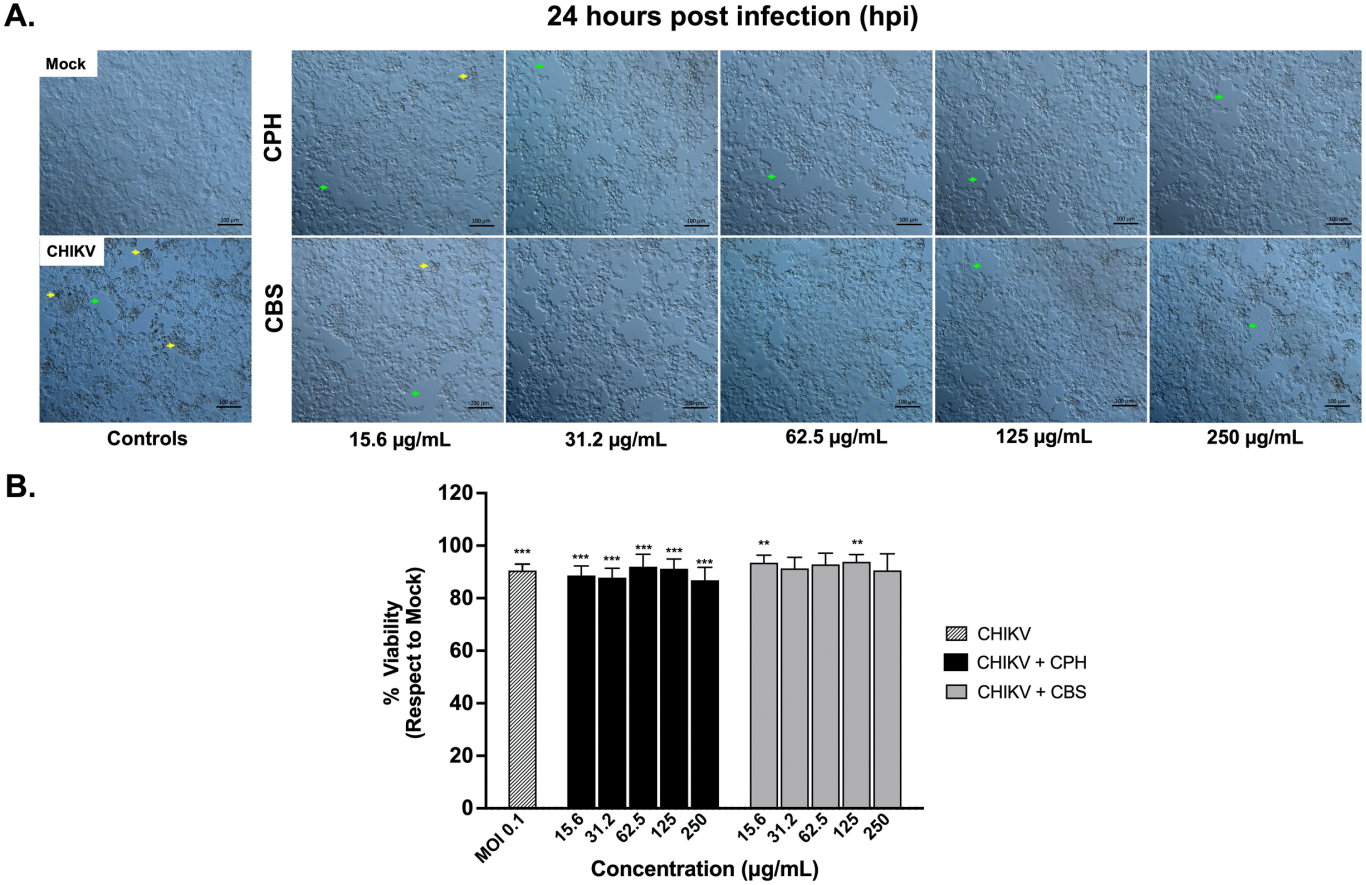
Cocoa by-products extracts attenuated morphological alterations in CHIKV-infected Huh-7 cells at 24 hpi. Huh-7 cells were infected with CHIKV at a MOI of 0.1 and then treated with 15.6, 31.2, 62.5, 125 and 250 µg/mL of CPH or CBS extracts. At 24 hpi the cells were analyzed to evaluate the cell morphology using phase-contrast microscopy (scale bar 100 µm) (A) or cell viability using a resazurin reduction assay (B). Untreated CHIKV-infected cells (CHIKV) and uninfected mock control cells (Mock) were included as experimental controls. Yellow arrows indicate virus-induced morphological alterations, whereas green arrows indicate cell detachment from the monolayer. The assays were performed in three independent experiments, each in triplicate. Results were reported as percentages of viability relative to the mock control and are expressed as the means ± SEM. Statistical significance was determined relative to the mock control (*p ≤ 0.05; **p ≤ 0.01; ***p ≤ 0.001).

By contrast, microscopic examination of CHIKV-infected Huh-7 cells revealed virus-induced morphological alterations that became more pronounced at 24 hpi. Prolonged viral exposure also resulted in progressive cell detachment from the monolayer (Figs 3A and 4A, Mock versus CHIKV). Notably, increasing concentrations of the CBS extract preserved cellular morphology and improved monolayer integrity at 24 hpi. Similarly, treatment with the CPH extract attenuated these cellular changes, maintaining Huh-7 cell morphology and adherence (Figs 3A and 4A).

Quantification of cell viability revealed that CHIKV-induced morphological changes were associated with a significant reduction in percentage cell viability at 12 and 24 hpi (Figs 3B and 4B). However, the decrease in metabolic activity in infected cells ranged from 4% to 10% relative to the mock control, indicating that overall viability remained above 80%. Similarly, Huh-7 cells infected and treated with either extract at all five concentrations also exhibited a significant reduction in the percentage of viable cells (maximum reduction of 13%); nevertheless, cell viability remained above 80% at both 12 and 24 hpi (Fig 3B and 4B).

Taken together, these findings suggest that cocoa by-products extracts did not induce marked cytotoxic effects within the tested concentration range, supporting their safety profile in Huh-7 cell culture. Consistently, comparable results were obtained in additional assays showing that CPH and CBS extracts, at concentrations up to 100 µg/mL and exposure times of 24, 48, and 72 h, neither altered cell morphology nor reduced metabolic activity below 80% in cancer-derived cell lines, including oral (CAL 27), lung (A549), colon (HT-29), and breast (MCF-7) cells (S1 Fig). Additionally, as expected, these extracts induce only a mild reduction in viability in the L929 cell line, a highly sensitive model commonly used in safety and biocompatibility assessments to detect potential cytotoxic effects (S1 Fig).

### Cocoa by-products extracts inhibit CHIKV replication

**Fig 5:**
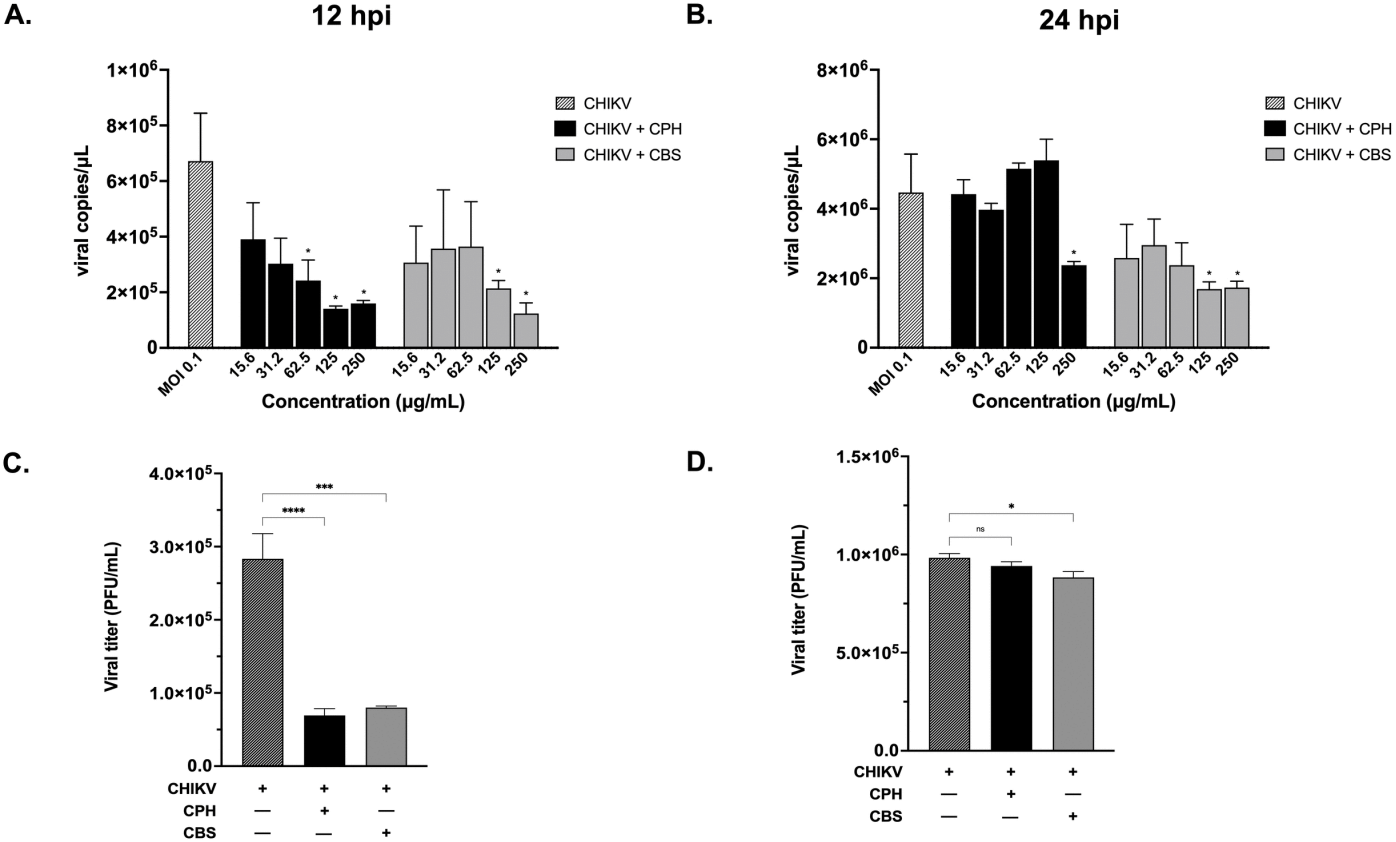
Cocoa by-products extracts inhibit CHIKV replication. Huh-7 cells were infected with CHIKV at a MOI of 0.1 and then treated with 15.6, 31.2, 62.5, 125 and 250 µg/mL of CPH or CBS extracts. At 12 hpi (A and C) or 24 hpi (B and D) supernatants were removed and analyzed to quantify viral RNA by RT-qPCR (A and B). Supernatants of CHIKV-infected Huh-7 cells treated with CPH or CBS extracts at 250 µg/mL were analyzed to quantify viral infectious particles by plaque assay using Vero cells (C and D). The assays were performed in three independent experiments, each in triplicate. Results are expressed as the means ± SEM. Statistical significance was determined relative to the CHIKV-infected cells (* p ≤ 0.05; ** p ≤ 0.01; *** p ≤ 0.001; **** p ≤ 0.0001).

After confirming that CHIKV-infected cells treated with cocoa extracts maintained their viability above 80%, we evaluated whether the extracts could inhibit viral replication at the previously tested concentrations. The results indicated that at 12 hpi the number of CHIKV genomic copies in the supernatants of cells treated with the highest concentrations of CBS extract (125 and 250 µg/mL) was significantly lower than in the untreated infected control (6.72 × 10⁵ copies/µL), with values of 2.14 × 10⁵ and 1.24 × 10⁵ copies/µL, respectively (Fig 5A). These reductions correspond to decreases of 68% and 81%, respectively, relative to the CHIKV-infected control.

Similarly, treatment with CPH extract at these concentrations, significantly reduced viral load to 1.41 × 10⁵ and 1.60 × 10⁵ copies/µL, corresponding to reductions of 79% and 76%, respectively (Fig 5A). In addition, cells treated with 62.5 µg/mL of CPH also exhibited a significant reduction of 64%, with 2.42 × 10⁵ copies/µL detected. At 24 hpi, a significant reduction in viral copies (47%) was observed only in the supernatants of cells treated with 250 µg/mL of CPH (2.38 × 10⁶ copies/µL). In contrast, treatment with CBS extract at 125 and 250 µg/mL, resulted in reductions of 62% and 61%, with viral loads of 1.69 × 10⁶ and 1.73 × 10⁶ copies/µL, respectively, compared to the untreated infected control (4.47 × 10⁶ copies/µL) (Fig 5B).

Furthermore, when supernatants from cells treated with the highest extract concentration (250 µg/mL) were used to infect Vero cell monolayers in a plaque assay, both CBS and CPH extracts significantly reduced plaque formation at 12 hpi, yielding titers of 8.00 × 10⁴ (71% reduction) and 6.92 × 10⁴ PFU/mL (75% reduction), respectively, compared with the control (2.83 × 10⁵ PFU/mL) (Fig 5C and S2 Fig). When Vero cell monolayers were infected with supernatants collected 24 hpi, only the CBS extract induced a significant, although modest, reduction in plaque formation (10%) with 8.83 × 10⁵ PFU/mL compared with 9.83 × 10⁵ PFU/mL in the control group (Fig 5D and S2 Fig). These findings indicate that both cocoa by-product extracts inhibit CHIKV replication *in vitro*, with a more pronounced antiviral effect during early stages of infection. Overall, the data support a dose-dependent antiviral activity of both extracts, with greater efficacy observed at earlier time points.

### The antiviral activity of extracts could be related to their cellular antioxidant activities

**Fig 6:**
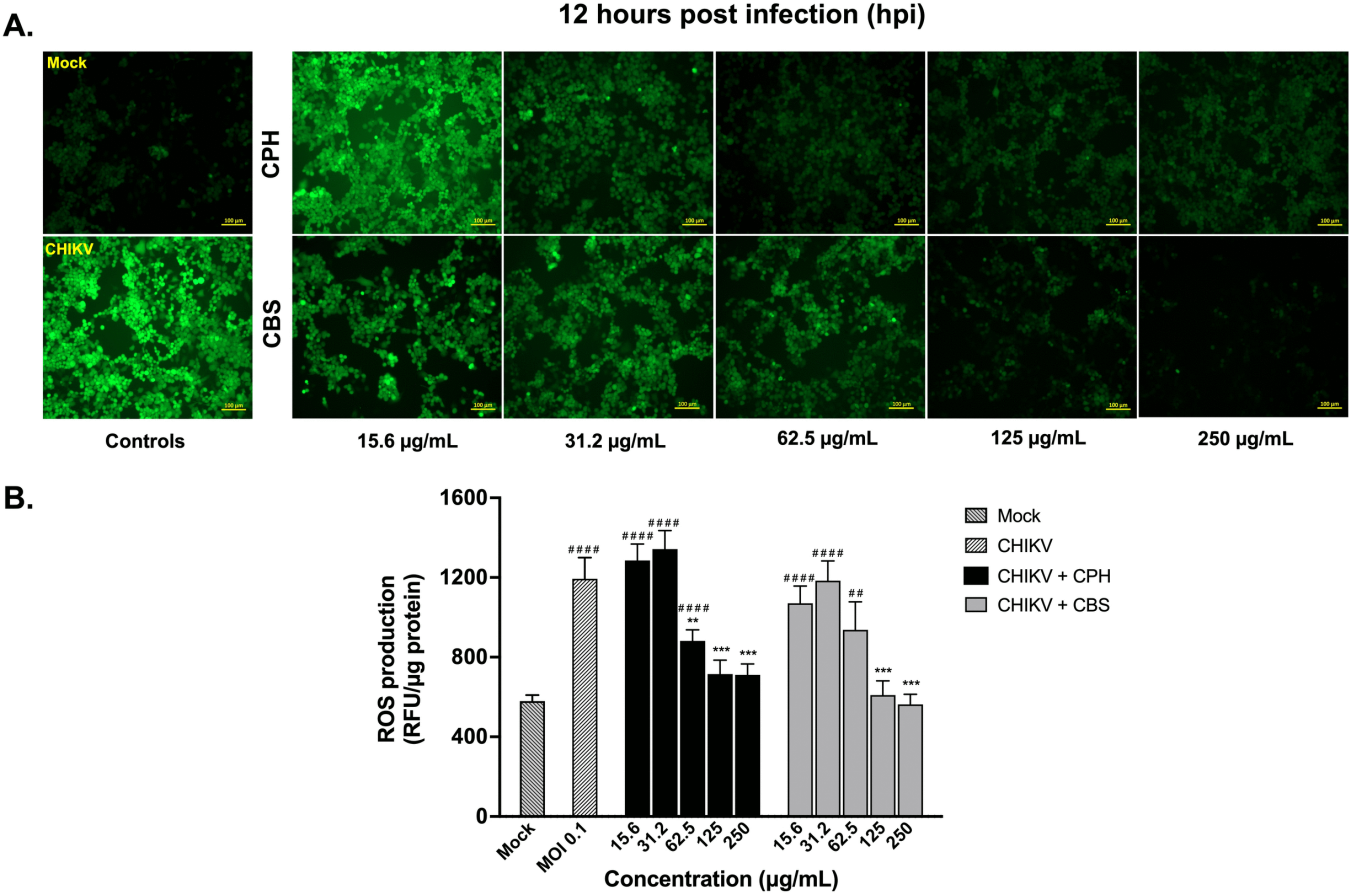
Cocoa by-products extracts exhibit cellular antioxidant activity in CHIKV-infected Huh-7 cells at 12 hpi. Huh-7 cells were infected with CHIKV at a MOI of 0.1 and then treated with 15.6, 31.2, 62.5, 125 and 250 µg/mL of CPH or CBS extracts. At 12 hpi the cells were analyzed to evaluate the total intracellular ROS levels using the DCFH-DA assay by fluorescence microscopy (scale bar 100 µm) (A) or using a spectrofluorometer (B). Untreated CHIKV-infected cells (CHIKV) and uninfected mock control cells (Mock) were included as experimental controls. The cellular ROS levels are reported as relative fluorescence units (RFU) per µg of protein. The assays were performed in three independent experiments, each in triplicate. Results are expressed as the means ± SEM. Statistical significance was determined relative to the CHIKV-infected cells (* p ≤ 0.05; ** p ≤ 0.01; *** p ≤ 0.001; **** p ≤ 0.0001) and mock control (^#^ p ≤ 0.05; ^# #^ p ≤ 0.01; ^# # #^ p ≤ 0.001; ^# # # #^ p ≤ 0.0001).

**Fig 7:**
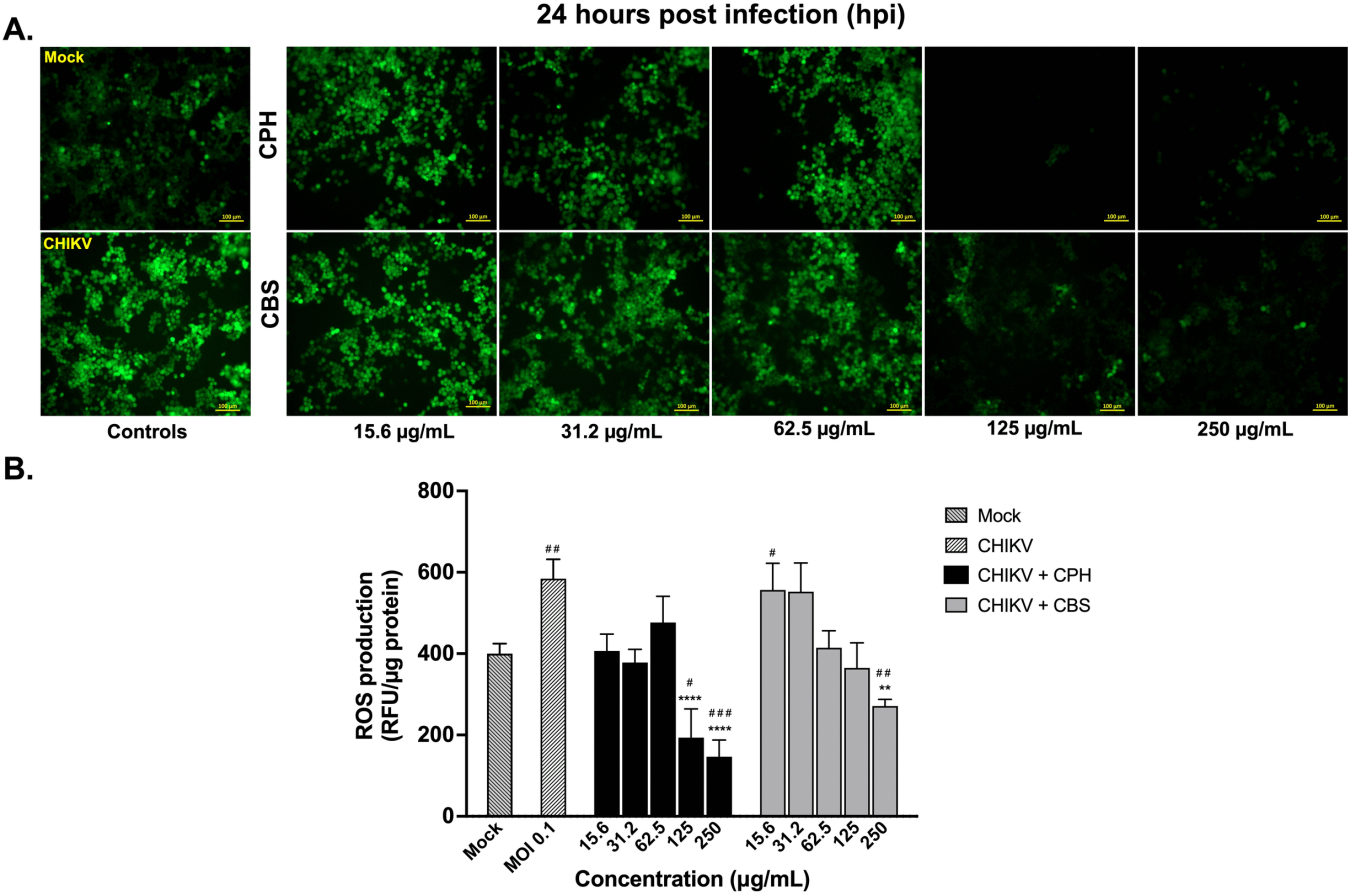
Cocoa by-products extracts exhibit cellular antioxidant activity in CHIKV-infected Huh-7 cells at 24 hpi. Huh-7 cells were infected with CHIKV at a MOI of 0.1 and then treated with 15.6, 31.2, 62.5, 125 and 250 µg/mL of CPH or CBS extracts. At 24 hpi the cells were analyzed to evaluate the total intracellular ROS levels using the DCFH-DA assay by fluorescence microscopy (scale bar 100 µm) (A) or using a spectrofluorometer (B). Untreated CHIKV-infected cells (CHIKV) and uninfected mock control cells (Mock) were included as experimental controls. The cellular ROS levels are reported as relative fluorescence units (RFU) per µg of protein. The assays were performed in three independent experiments, each in triplicate. Results are expressed as the means ± SEM. Statistical significance was determined relative to the CHIKV-infected cells (* p ≤ 0.05; ** p ≤ 0.01; *** p ≤ 0.001; **** p ≤ 0.0001) and mock control (^#^ p ≤ 0.05; ^# #^ p ≤ 0.01; ^# # #^ p ≤ 0.001; ^# # # #^ p ≤ 0.0001).

Microwave assisted extraction is a green technology that yields extracts with high antioxidant capacity [28]. Given that virtually no studies have evaluated the antiviral effect of CPH and CBS extracts against CHIKV in Huh-7 cells and considering that CHIKV replication is linked to an increased (ROS) production, we sought to determine whether the antiviral activity of the extracts is related to their antioxidant properties. As shown in Fig 6A, fluorescence microscopy analysis suggests that ROS production was elevated in CHIKV-infected Huh-7 cells compared to the basal state (Mock). As expected, treatment with CPH and CBS extracts led to a reduction in ROS levels in CHIKV-infected cells, an effect that was more pronounced at higher concentrations (125 and 250 µg/mL) and was evident at both 12 and 24 hpi. (Fig 6A and 7A).

Spectrofluorometric analysis revealed significantly higher ROS production in CHIKV-infected cells compared with the mock control at both 12 and 24 hpi. In addition, treatment with the extracts resulted in a reduction in relative fluorescence units (RFUs), indicating decreased ROS production (Fig 6B and 7B). In infected cells treated with the CBS extract, a statistically significant reduction in ROS levels was observed compared with the untreated infected control (1193.4 RFU/µg protein at 12 hpi). Specifically, CBS at 125 and 250 µg/mL reduced ROS levels by 49% and 53%, respectively (609.3 and 562.9 RFU/µg protein), reaching values comparable to the mock control (579.4 RFU/µg protein) (Fig 6B). At 24 hpi, treatment with 250 µg/mL of CBS further decreased ROS levels to 271.3 RFU/µg protein (54% reduction), significantly lower than both the untreated infected control (584.3 RFU/µg protein) and the mock control (399.9 RFU/µg protein) (Fig 7B).

Similarly, the CPH extract significantly reduced ROS production at 12 hpi by 26%, 40%, and 40% at concentrations of 62.5, 125, and 250 µg/mL, respectively (881.5, 715.2, and 710.9 RFU/µg protein), compared with the untreated infected control (1193.4 RFU/µg protein) (Fig 6B). However, treatment with 62.5 µg/mL of CPH maintained ROS levels significantly higher than those of the mock control. At 24 hpi, CPH at 125 and 250 µg/mL reduced ROS levels to 193.5 RFU/µg protein (67% reduction) and 146.7 RFU/µg protein (75% reduction), respectively, values significantly lower than those observed in both the untreated infected control and the mock control (Fig 7B).

Collectively, these findings suggest a strong association between the antioxidant capacity of the extracts and their antiviral activity, demonstrating a concentration-dependent effect reflected by reductions in both viral genome copies and ROS levels. Consistent with the plaque assay data, this biological effect was more pronounced at 12 hpi.

## Discussion

Although a vaccine for chikungunya has been available since November 2023 and approved by the U.S. Food and Drug Administration (FDA) for individuals over 18 years of age [29], its distribution remains limited in regions where the vector is established and active CHIKV transmission persists. In 2025, from epidemiological week (EW) 1 through EW 53, a total of 313,132 chikungunya cases were reported by 18 countries and one territory in the Americas, including 113,926 laboratory-confirmed cases and 170 deaths attributed to the infection [30]. Furthermore, climate models predict an increase in mean annual temperatures due to global warming, particularly in regions such as southern Europe [31,32], which could facilitate the spread of arbovirus-related diseases, including CHIKV [33]. This scenario underscores the need for complementary antiviral strategies to manage the disease, particularly in populations lacking prior immunity to the virus. In this context, and to our knowledge, this is the first report describing the effects of CPH and CBS extracts on CHIKV replication. Beyond their biological relevance, these findings also expand the potential pharmaceutical valorization of organic by-products generated during the cocoa processing.

Given that oxidative stress and inflammatory signaling contribute to CHIKV pathogenesis and viral replication dynamics, the antioxidant and anti-inflammatory properties of cacao-derived metabolites may represent relevant biological mechanisms underlying the antiviral effects observed. Although the present study was not designed to dissect specific molecular pathways, our results show that modulation of intracellular redox balance could influence viral replication efficiency. This effect may be related to the presence of metabolites with previously documented biological activity. The chemical composition of both extracts comprises a combination of compounds with well-documented antioxidant, anti-inflammatory, and, in some cases, direct antiviral properties, which could collectively contribute to the biological responses observed in CHIKV-infected cells. Cocoa-derived matrices rich in alkaloids such as caffeine and theobromine, as well as phenolic compounds, have been shown to reduce intracellular ROS levels and downregulate inflammatory mediators including NO, PGE2, TNF-α, MCP-1, and IL-6 in cellular models [14]. Importantly, caffeine has been reported to inhibit CHIKV replication *in vitro* under both pre- and post-infection treatment conditions [34], providing direct evidence that at least one major alkaloid present in these extracts may exert anti-CHIKV activity. The detection of fatty acid–derived metabolites such as oleoylethanolamide (OEA) further supports the antioxidant framework. OEA supplementation has been associated with improved oxidative stress biomarkers in human studies, including enhanced antioxidant defenses and reduced lipid peroxidation [35]. Likewise, branched-chain amino acids (BCAAs), including valine and isoleucine, may influence immune competence and protein synthesis through mTOR signaling pathways [36], while also modulating the NAD ⁺ /NADH balance and limiting ROS accumulation [37,38]. Other amino acids identified, including proline, tryptophan, phenylalanine, and tyrosine, have been associated with enhanced cellular survival under oxidative stress conditions. Proline, in particular, has been shown to preserve intracellular redox balance by maintaining the GSH/GSSG ratio and reducing ROS levels under oxidative challenge [39]. Ferulic acid, a major phenylpropanoid detected in the CPH extract, is widely recognized for its antioxidant, anti-inflammatory, antimicrobial, and emerging antiviral activities. Mechanistically, it has been shown to enhance type I interferon responses, promote heme oxygenase-1 (HO-1) expression, and suppress the production of inflammatory mediators such as MIP-2 and IL-8 during viral infections [40]. Collectively, these metabolites provide a plausible biochemical basis supporting the antioxidant and potential antiviral responses observed in this study.

Regarding cellular effects, CHIKV infection induced characteristic cytopathic alterations in Huh-7 cells, including cell rounding and monolayer detachment, consistent with previous reports [41,42]. Despite this morphological damage, infected cells retained metabolic activity, as evidenced by the reduction of resazurin to resorufin by cellular oxidoreductases [43]. Several mechanisms could explain this phenomenon. For instance, various viruses, including CHIKV, have been shown to manipulate host cell metabolism to sustain active viral replication [44,45]. Additionally, positive-sense RNA viruses can reorganize intracellular membranes to form viral replication complexes, which may contribute to morphological damage [46]. CHIKV has also been reported to induce autophagy, promoting its replication while avoiding cell death [46,47].

In relation to the observed loss of adherence, some cells detached during washing steps performed in antioxidant assays; therefore, fluorescence data were normalized to total protein content in each well. Cytopathic effects such as these are commonly associated with viral manipulation of the actin and tubulin cytoskeleton during assembly and release [48,49], as well as interference with adhesion molecules including integrins and cadherins [50]. In the case of CHIKV, activation of pro-inflammatory pathways can influence cytoskeletal reorganization and interactions with the extracellular matrix [51]. Interestingly, treatment with CPH and CBS extracts partially mitigated these cytopathic alterations in a concentration-dependent manner, preserving morphological integrity and monolayer confluence, suggesting a cytoprotective effect.

Compounds such as flavonoids and alkaloids play a crucial role in reducing oxidative stress and enhancing cellular defense mechanisms against high ROS levels thereby minimizing structural damage [13,52–54]. This protective effect may explain the cytoprotective properties observed in the present study. Qualitative and quantitative analysis showed a marked reduction in DCF fluorescence at concentrations ≥125 µg/mL, especially at 12 hpi, when ROS accumulation appeared to peak. At 24 hpi, fluorescence intensity decreased, possibly reflecting activation of endogenous antioxidant responses, while extract-mediated antioxidant effects remained detectable. Interestingly, while CBS maintained its inhibitory effect over time, the antioxidant efficacy of CPH was reduced at 24 hpi. This difference may be related to variations in the chemical stability of their bioactive constituents. As detailed in Tables 2 and 3, HPLC-ESI-QTOF-MS analysis revealed a higher relative abundance of flavonoids in CPH, whereas CBS was characterized by a predominance of xanthine alkaloids, including caffeine and theobromine. Flavonoids are known to be susceptible to degradation under certain environmental conditions [55–57], while xanthine derivatives generally exhibit greater structural stability [55]. Nevertheless, this interpretation remains speculative and requires further analytical validation. Although both extracts reduced ROS levels and viral genome copies in Huh-7 cells, the present data do not establish a direct causal relationship between antioxidant modulation and antiviral activity. Further studies are required to elucidate the specific molecular pathways involved in CHIKV inhibition.

## Conclusion

This study demonstrated that CPH and CBS extracts effectively inhibited viral replication in CHIKV-infected Huh-7 cells. Interestingly, CBS demonstrated greater antiviral potential, as its inhibitory effects were most evident at the highest tested concentrations at both 12 and 24 hpi. On the other hand, CHIKV infection was found to induce oxidative stress in Huh-7 cells by increasing the production of reactive oxygen species (ROS). This effect was mitigated by both extracts in a dose-dependent manner. Notably, the antioxidant properties of these extracts were sustained for up to 24h of exposure. Finally, while the antiviral activity of cacao by-products extracts may be associated with their cellular antioxidant effects, further studies are required to elucidate their precise mechanism of action in inhibiting viral replication.

## Supporting information

S1 File Supplementary Materials and methods and Supplementary Figure legends. (DOCX)

S1 Fig Cocoa by-products extracts did not exhibit any cytotoxic effects on human tumor cell lines and fibroblast L929. (TIF)

S2 Fig Antiviral activity of CPH and CBS extracts against CHIKV assessed by plaque assay in Vero cells. (TIF)
