# Supplementary material for: Cocoa by-products extracts suppress viral replication and oxidative stress in chikungunya virus-infected cells
Source: PLoS One. 2026 Jul 22;21(7):e0354240. doi: 10.1371/journal.pone.0354240 (PMC13390854; doi:10.1371/journal.pone.0354240)
Supplement: S1 File — (DOCX) [file pone.0354240.s001.docx]

**Supplementary Materials and methods**

**Cell lines and Growth Conditions**

The cytotoxic potential of the extracts was evaluated *in vitro* using the tumor cell lines: HT-29 (ATCC HTB-38; CVCL_0320), a human colorectal adenocarcinoma cell line derived from a 44-year-old Caucasian female; CAL-27 (ATCC CRL-2095; CVCL_1107), a human tongue squamous cell carcinoma cell line derived from a 56-year-old male; and MCF-7 (ATCC HTB-22; CVCL_0031), a breast cancer cell line isolated from the pleural effusion of a 69-year-old Caucasian woman. To assess the extracts' selectivity and safety, additional analyses were conducted on L929 mouse fibroblast (ATCC CCL-1; CVCL_0462) a cell line derived from subcutaneous tissue of *Mus musculus*, widely used in cytotoxicity and biocompatibility studies. The cells were cultured in DMEM medium (UltraCruz™) supplemented with 1 mM sodium pyruvate, 25 mM HEPES, 100 U/ml penicillin, 100 µg/ml streptomycin, and 10% heat-inactivated fetal bovine serum (Gibco BRL). Cells were propagated in T-75 cm² culture flasks at 37°C in a 5% CO_2_ atmosphere with 90% relative humidity.

**Cell viability assay**

The viability of tumor cells and L929 was assessed by resazurin reduction assay. Briefly, cells in 80% confluence were detached, counted, and seeded into 96-well flat-bottom plates at the following densities per well: 20,000 for HT-29, 10,000 for CAL-27, and 5,000 for MCF-7, A549, and L929 cells. After 24 h, the cells were exposed to five serial 1:2 dilutions of the extracts (ranging from 100 to 6.25 µg/mL) for 24, 48, and 72 h. Paclitaxel (100 µM) served as death control, and the vehicle’s effect was assessed in all assays. After the exposure periods, the medium was replaced with 100 μL of 44 μM resazurin. Following a 4-h incubation, fluorescence from viable/metabolically active cells was measured using a TECAN GENios spectrofluorometer (excitation: 535 nm, emission: 595 nm). Assays were performed over three independent weeks in triplicate, and results were expressed as percentage viability relative to the vehicle control.

**Supplementary Figure legends**

**S1 Fig. Cocoa by-products extracts did not exhibit any cytotoxic effects on human tumor cell lines and fibroblast L929.** Cells were treated with 6.25, 12.5, 25, 50 and 100 µg/mL of CPH or CBS extracts and incubated for 24, 48 and 72 h. Then, cells lines from oral cancer (A), lung cancer (B), colon cancer (C), breast cancer (D) and normal fibroblasts (E) were analyzed to evaluate the cell viability using a resazurin reduction assay. The assays were performed in three independent experiments, each in triplicate. Results were reported as percentages of viability relative to the vehicle control and are expressed as the means ± SEM. Statistical significance was determined relative to the vehicle control (* p ≤ 0.05; ** p ≤ 0.01).

**S2 Fig.** **Antiviral activity of CPH and CBS extracts against CHIKV assessed by plaque assay in Vero cells.** Serial dilutions of culture supernatants collected at 12 and 24 hpi from CHIKV-infected Huh-7 cells treated with CPH (left panels) or CBS (right panels) extracts were subjected to plaque assay in Vero cells to determine viral titers (PFU/mL). The images show three representative replicates per condition of plaques formed during the assay. The dilution factor is indicated at the bottom of each image.
